# Hepatitis C Virus Core Protein Inhibits Interferon Production by a Human Plasmacytoid Dendritic Cell Line and Dysregulates Interferon Regulatory Factor-7 and Signal Transducer and Activator of Transcription (STAT) 1 Protein Expression

**DOI:** 10.1371/journal.pone.0095627

**Published:** 2014-05-01

**Authors:** Amy E. L. Stone, Angela Mitchell, Jessica Brownell, Daniel J. Miklin, Lucy Golden-Mason, Stephen J. Polyak, Michael J. Gale, Hugo R. Rosen

**Affiliations:** 1 Integrated Department in Immunology, University of Colorado Denver and National Jewish Health, Denver, Colorado, United States of America; 2 Division of Gastroenterology & Hepatology, Hepatitis C Center, Department of Medicine, University of Colorado Denver, Aurora, Colorado, United States of America; 3 Department of Global Health, University of Washington, Seattle, Washington, United States of America; 4 Department of Laboratory Medicine, University of Washington, Seattle, Washington, United States of America; 5 Department of Immunology, University of Washington, Seattle, Washington, United States of America; 6 Denver Veteran’s Affairs Medical Center, Denver, Colorado, United States of America; Saint Louis University, United States of America

## Abstract

Plasmacytoid Dendritic Cells (pDCs) represent a key immune cell population in the defense against viruses. pDCs detect viral pathogen associated molecular patterns (PAMPs) through pattern recognition receptors (PRR). PRR/PAMP interactions trigger signaling events that induce interferon (IFN) production to initiate local and systemic responses. pDCs produce Type I and Type III (IFNL) IFNs in response to HCV RNA. Extracellular HCV core protein (Core) is found in the circulation in chronic infection. This study defined how Core modulates PRR signaling in pDCs. Type I and III IFN expression and production following exposure to recombinant Core or β-galactosiade was assessed in human GEN2.2 cells, a pDC cell line. Core suppressed type I and III IFN production in response to TLR agonists and the HCV PAMP agonist of RIG-I. Core suppression of IFN induction was linked with decreased IRF-7 protein levels and increased non-phosphorylated STAT1 protein. Circulating Core protein interferes with PRR signaling by pDCs to suppress IFN production. Strategies to define and target Core effects on pDCs may serve to enhance IFN production and antiviral actions against HCV.

## Introduction

Hepatitis C Virus (HCV) is a major public health problem infecting approximately 200 million people globally [1], leading to persistence in over 80% of cases. Infection is initially sensed by the host innate immune response which leads to activation of adaptive immunity. Two major pattern recognition receptor (PRR) systems can recognize the presence of viral infection to mount an immune response: Toll-like receptors (TLRs) are endosomal pattern recognition receptors (PRR); and retinoic acid inducible gene-I (RIG-I)-like receptors (RLRs) were identified as cytosolic receptors for intracellular dsRNA sensing [2], [3]. The relative contribution of TLRs and RLRs as viral sensors varies between viruses and cell types [2].

Immune modulation is a common viral immune evasion strategy and HCV is no exception. The nucleocapsid protein, also known as the HCV core protein (Core), functions as a structural protein, a mediator of genome packaging, and an immune modulator [4], [5]. Core has been reported to modulate the immune response at multiple levels, including the innate hepatocyte response [6] and T cells through binding to gC1qR [7], the globular region of complement protein 1q receptor. The precise interaction between Core and host proteins has been studied by various groups [5], showing pathways mediated through interactions with TLR2, TLR4 and gC1qR [8], [9].

Plasmacytoid dendritic cells (pDCs) are a rare population of leukocytes whose key function is to detect and respond to viruses [10]. These cells use PRRs, primarily TLRs, to detect nucleic acids from viral infections [11]. pDCs produce all Type I (IFNα/β) and III (IFNλ) IFNs following stimulation with synthetic TLR ligands [12] and are responsive to IFNα/λ [13], [14]. Interferon regulatory factor (IRF)-7 is constitutively expressed in pDCs and use a Myeloid Differentiation primary response protein 88 (MyD88)- and IRF-7-dependent pathway for production of Type I IFNs [15]. Contrary to most other cell types, active viral replication is unnecessary to induce the production of IFNs by pDCs [16].

Accumulating evidence suggests that HCV targets pDCs to control the host antiviral response. Indeed, HCV infection has been associated with depletion and functional suppression of pDCs [17], [18], [19], [20]. Moreover, the HCV E2 glycoprotein modulates pDC function through binding of BDCA-2 to suppress IFN signaling [20]. Although pDCs recognize HCV RNA and release IFNα/β/λ to control viral replication in hepatocytes [21], [22], [23], it is unclear whether HCV has developed additional mechanisms to disrupt the pDC response. We hypothesized that Core, which circulates at high concentrations in the serum of infected patients [24], attenuates IFN responses from pDCs. Using the GEN2.2-pDC line, we found that both TLR- and RLR-mediated IFN responses were decreased by Core. This effect was associated with decreased IRF-7 and increased non-phosphorylated (i.e. inactive) STAT1.

## Materials and Methods

### Cell Culture

GEN2.2 cell line were grown as previously described [21], [25]. For experiments, the non-adherent BDCA-2+ and CD45+ fraction of the culture was used.

### Core Pretreatment

Cells were plated at 1×10^6^ cells/mL and treated with 10 µg/mL rCore or β-galactosidase (Virogen) [26] for 24 hours, then washed twice in PBS and replated.

### TLR Stimulation

TLR stimulation occurred as previously described [21]. Briefly, following rCore pretreatment, TLR ligands or media were added: ODN 2216 CpG [250 µM, Invivogen tlrl-hodna], Loxiribine [1 mM, Invivogen tlrl-lox]. The cells were incubated for 6 hours then RNA was isolated and cDNA was made. qRT-PCR was performed using SYBR Green primers and master mix (Qiagen) and run on a StepOnePlus qPCR machine (Applied Biosystems). Data was analyzed by the ΔΔCT method.

### HCV PAMP Stimulation

Following Core pretreatment, 0.5 µg of pU/UC, or X-region RNA (prepared as previously described [21]) were transfected (Mirus 2250) for 2, 4, 8 or 24 hours. RNA was isolated, cDNA was generated and qRT-PCR was run and analyzed as described for TLR stimulation. Supernatants were also collected at 2, 4, 8 or 24 hours post-transfection.

### ELISAs

ELISA kits for IFNα (PBL Interferon Source), IFNβ (PBL Interferon Source) and IL-29/IFNλ1 (eBiosciences) were used as per the manufacturer’s instructions. All samples used at either a 1∶1 or 1∶10 dilution and were incubated overnight at 4C.

### IFNβ Promoter-luciferase Reporter

Cells were plated and stimulated with rCore or β-gal as described above. Along with pU/UC RNA, the pIFN-β-luc (2 ug) and the pCMV-*Renilla* -luc (400 ng) were transfected using Mirus 2250 as described above. After 24 hours, the luciferase activity was measured using the Dual-Luciferase reporter assay system (Promega). All conditions and experiments were conducted in triplicate. The plasmids pIFN-β-luc and pCMV-*Renilla*-luc have been previously described [27].

### LPS Stimulation

Cells were pretreated with 10 ng/mL of LPS (Sigma) for 24 hours then washed twice in DPBS and stimulated with HCV pU/UC RNA as described above.

### Flow Cytometry

Cells were washed with FACS wash (PBS with 0.016% sodium azide, 0.6% BSA) and then resuspended in FACS wash containing fluorescently labeled antibodies (BD Biosciences) and incubated at 4C for 30 minutes. After washing twice in FACS wash cells were resuspended in 2% PFA. Cells were acquired on BD FACSCanto II. Data was analyzed using FlowJo software. Apoptosis Staining: Cells were fixed in 4% PFA for 20 min. After washing with FACS Wash, cells were resuspended in binding buffer and Anti-Annexin V antibody (BD Biosciences) and incubated 15 min. Following quenching with binding buffer, 7-AAD (5 µg/mL final) was added to the cells and incubated for 15 min. Cells were acquired immediately. CFSE: Cells were resuspended in PBS+0.1% BSA at 10^7^ cells/mL and CFSE (Carboxyfluorescein succinimidyl ester) was added to a final concentration of 1 µM. Cells were incubated at 37°C for 10 min. Following quenching, cells were washed three times with RPMI+10% FBS and resuspended in appropriate media and cultured as required. Phosphoflow: Cells were stimulated with or without protein as described for Core stimulations and fixed at 0, 5, 15, 30 or 60 min overnight. Cells were washed twice and resuspended in BD Perm Buffer III then washed as before. Cells were then incubated with antibodies (STAT1 n-terminus, STAT1pY701, STAT1pS727, pERK1/2, pAKT, STAT3pS727, IRF7, pIRF7: BD Biosciences). Cells were then washed twice and acquired.

### Immunofluorescence

Cells were stimulated and fixed. Cells were cytospun on slides (Shandon Cytospin 2 Cat #59900102) and dried at RT. Slides were not mounted and shipped to the University of Washington for further processing and imaging. Following re-hydration in PBS, cells were blocked with 3% normal goat serum (Jackson ImmunoResearch). Primary antibody staining was performed with rabbit anti-STAT1 (Santa Cruz Biotechnology, sc-346), anti-IRF7 (Santa Cruz Biotechnology) or mouse anti-HCV Core (Thermo Scientific MAB1-080). Secondary antibody staining was performed with goat anti-rabbit or goat anti-mouse conjugated to AlexaFluor488 (Santa Cruz Biotechnology). Nuclei were stained with DAPI. Slides were mounted using ProLong Gold Anti-Fade Reagent (Invitrogen). Images were captured at 40x magnification using the EVOS FL Cell Imaging System (Invitrogen).

### IFNα Stimulation

Cells were pretreated with Core or β-galactosidase for 24 hours as described above then stimulated with 100 ng/mL of recombinant IFNα2a (Hoffman Roche). Cells were harvested and assayed as described.

### Western Blots

Cell lysates were prepared as previously described [21] following stimulation as described above. Samples were separated using SDS-PAGE on Mini-protean TGX Any kD gels (Bio-rad) and transferred onto a nitrocellulose membrane using a wet transfer system. Membranes were blocked, washed, and proteins were analyzed by immunoblotting with standard methods using antibodies specific to IRF-7, IRF-3 (both from Cell Signaling Technology) and GAPDH (Abcam). Secondary antibodies conjugated to HRP were obtained from Jackson ImmunoResearch and immunoreactive bands were detected with the Immuno-Star HRP Substrate kit (Bio-Rad). For time courses, cells were stimulated as described above and lysed directly in Laemmli buffer (Bio-rad) and equal volumes were assayed as with other western blots using antibodies specific to STAT1 and pSTAT1Y701 (Abcam). Densitometry was performed using ImageJ (NIH), and proteins of interest were normalized to a reference protein (GAPDH).

### Statistics

Statistics were performed using Graphpad Prism statistical package. Mann-Whitney non-parametric test was used for comparisons amongst groups. One sample t-tests or Mann-Whitney tests were used to compare fold increases of stimulated conditions with control conditions.

## Results

### Core Inhibits TLR and RLR Stimulated IFN Production

**Figure 1:**
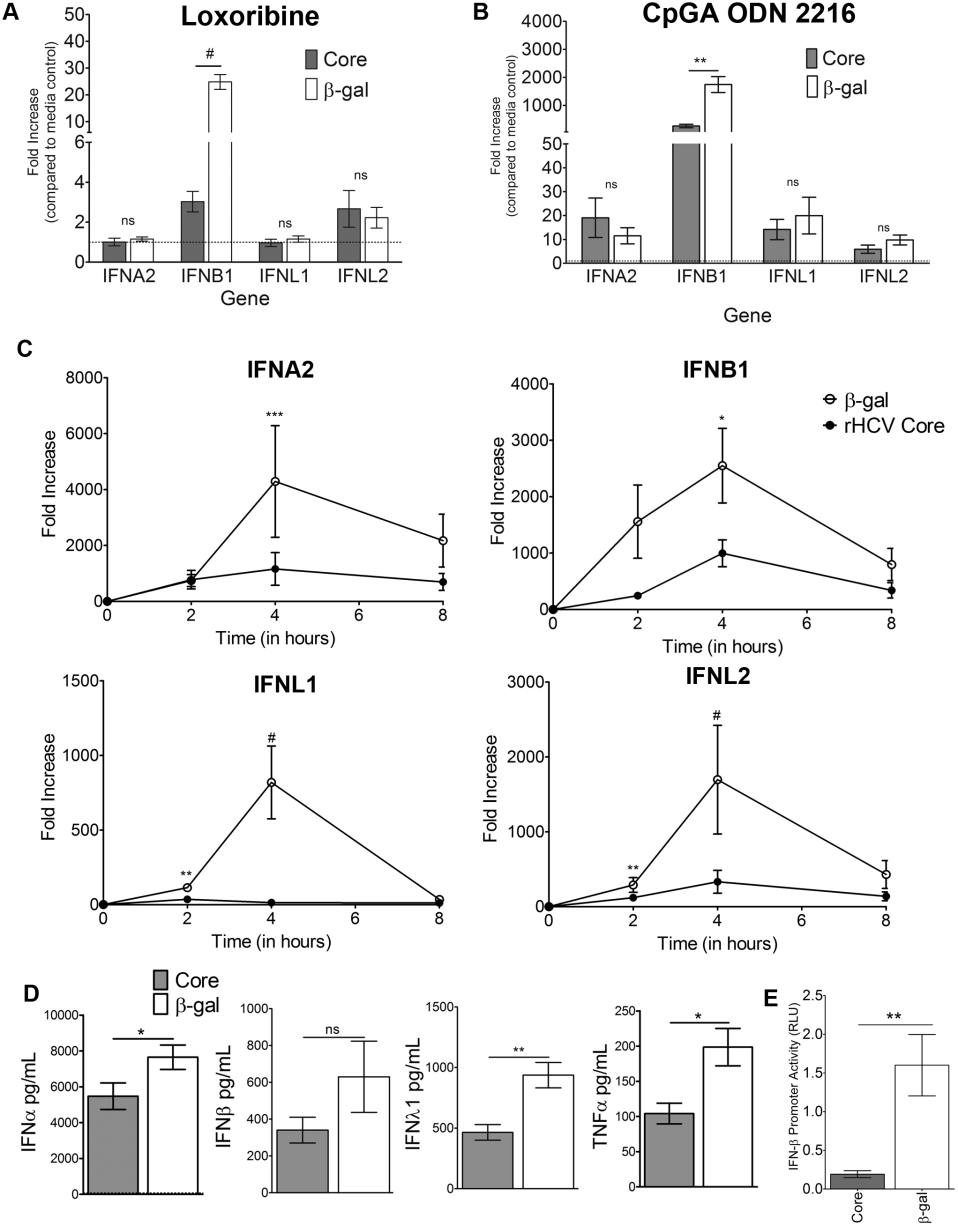
rCore inhibits TLR stimulation. Gene fold increases in GEN2.2-pDCs following rCore pretreatment and TLR stimulation with Loxoribine (A) or CpGA (B). C) Kinetics of IFN mRNA after pretreatment with rCore or β-galactosidase (β-gal) and HCV pU/UC RNA stimulation. D) Levels of protein by ELISA when cells were treated with rCore or β-gal for 24 h then stimulated with the HCV PAMP RNA for 24 h. E) IFNβ promoter activity following 24 hours of rCore/β-gal pretreatment and 24 hour transfection of pU/UC RNA and IFNβ –firefly luciferase reporter plasmid. Units are shown as Relative Light Units (RLU) and represent the light units measured of firefly luciferase (driven by the IFNβ promoter) divided by the light units of renilla luciferase (transfection control; driven by the CMV promoter). Combined data for 3 (A, B, D & E) or 8 (C) independent experiments. P values are results of Mann-Whitney comparison of the bars indicated. *p<0.05 **p<0.01 ***p<0.001 #p≤0.0001. Mean +/− SEM.

It has been well established that pDCs respond to viral RNA analogues through TLR pathway activation and more recently, RLR pathway activation [21]. In order to determine if Core disrupts TLR signaling we pretreated GEN2.2-pDCs [25] with recombinant HCV Core protein (rCore) followed by stimulation with Loxoribine (TLR7 agonist) or Type A CpG ODN2216 (TLR9 agonist; CpGA). Compared to the control protein (β-galactosidase), pretreatment of GEN2.2-pDCs with rCore led to statistically lower IFNB1 mRNA production following Loxoribine (**Figure 1A**) or CpGA (**Figure 1B**). Other Type I and III IFNs were not influenced by rCore pretreatment. These data suggest that Core imparts differential effects on PRR signaling pathways leading to IFN induction.

Over the course of 24 hours, rCore inhibited IFN production induced by the RIG-I ligand HCV PAMP, i.e., pU/UC tract RNA ([28], [29]; **Figure 1C**). At 4 hours, the peak of responses, rCore pretreatment significantly decreased IFNα/β/λ mRNA. At the protein level, TNFα, IFNα, and IFNλ1 production was diminished by rCore pretreatment (**Figure 1D**). Furthermore, the activity of the IFNβ promoter was significantly decreased in the rCore treatment as compared to the β-gal treatment as measured by an IFNβ promoter-luciferase reporter system (**Figure 1E**).

### Core does not Significantly Affect Basal IFN Levels, Cell Death or Proliferation

Stimulation with rCore alone did not lead to reduced IFN gene expression and only modest increase in IFNB1 and SOCS3 mRNA expression (**Figure S1A**) in resting pDCs. LPS treatment did not inhibit pU/UC RNA induced IFN production (**Figure S1B**), thus eliminating the possibility of endotoxin contamination in the rCore preparations as the reason for IFN mRNA inhibition. To eliminate the possibility that cell death was the cause of the reduced IFN responses, we determined the ability of rCore to induce apoptosis in the GEN2.2-pDC line. Treatment with rCore did not induce apoptosis above that caused by the presence of β-galactosidase control protein (**Figure S1C**). To examine the effect of rCore on the proliferation of GEN2.2-pDCs, we stained the cells with CFSE and treated the cells with rCore before harvesting over various time points. We observed that rCore does not inhibit proliferation of GEN2.2-pDCs (**Figure S1D**). Moreover, rCore was visualized by immunofluorescence microscopy within the GEN.2.-pDCs, thereby confirming exposure and presence of rCore associated with the cells (**Figure S1E**).

### rCore Induces Differential Expression of IFN Transcription Factors and IFN Signaling Factors Following Activation of the RLR Pathway

IFN regulatory factors (IRFs) are critical transcription factors that drive PRR-mediated expression of IFNs and antiviral genes [30]. In particular, IRF-3 and IRF-7 regulate Type I IFN responses after RNA virus infection [31], [32]. Activation of IRF-3 is essential for IFN production from hepatocytes during HCV RNA replication [27], [33] whereas in West Nile Virus infection, IRF-7 has been demonstrated to be critical for Type I IFN production and viral control [34], [35].

**Figure 2:**
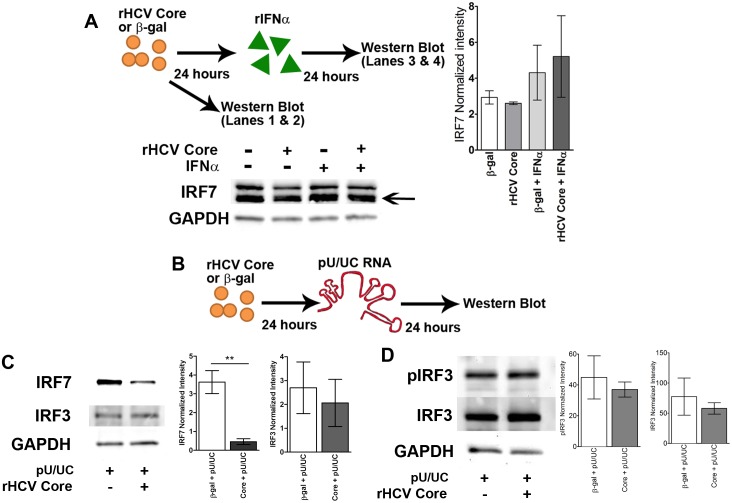
rCore changes transcription factors related to IFN production. A) Top – Schematic of experimental design. Western blot of IRF-7 following 24 h of rCore/β-gal treatment (lanes 1 & 2) and 24 h of rCore/β-gal treatment followed by 24 h of IFNα (100 ng/mL) treatment (lanes 3 & 4). Arrow indicates IRF-7 band of interest. B) Schematic of experimental design. C) Western blots of IRF-7 and IRF-3 following 24 h of rCore/β-gal treatment and 24 h of HCV PAMP RNA treatment. Densitometry for IRF-7 (left) and IRF-3 (right). D) Western blots of IRF-3 and IRF-3pS396 following 24 h of rCore/β-gal treatment and 24 h of HCV PAMP RNA treatment. Densitometry for IRF-3 (left) and pIRF-3 (right). Graphs show combined densitometry data after normalization to the loading control for 3 independent experiments. Images are representative blots. P values are results of Mann-Whitney comparison of the bars indicated. **p<0.01. Mean +/− SEM.

Accordingly, we examined the effect of rCore on signaling by IRFs. Treatment with rCore or β-gal did not change the amounts of IRF-7 present in the GEN2.2-pDCs (**Figure 2A** **, lanes 1 & 2**). Upon subsequent treatment with IFNα IRF-7 levels increased equally regardless of the protein exposure (**Figure 2A** **, lanes 3 & 4**). When we examined IRF-7 and IRF-3 levels following 24 hours of protein treatment prior to 24 hours of pU/UC RNA stimulation (Schematic of experimental design, **Figure 2B**) we found that rCore reduced IRF-7 but not IRF-3 protein levels (**Figure 2C**). Furthermore, the levels of IRF-7 did not change during the first hour of pU/UC stimulation regardless of protein pretreatment (**Figure S2**). These data suggest that rCore prevents IRF-7 induction by IFN, while IRF-3 is unaffected. Using Western Blotting, we examined the phosphorylation levels of IRF-3. As we previously found, total IRF-3 levels remain unchanged after treatment (**Figure 2D**). Additionally, phospho-IRF-3 levels were unchanged. Our results are in accord with the observation that in pDCs, IRF-7 is a master regulator of IFN production, while IRF-3 is dispensable for IFNα production [15].

### rCore Alters STAT-1 Proteins Levels

**Figure 3:**
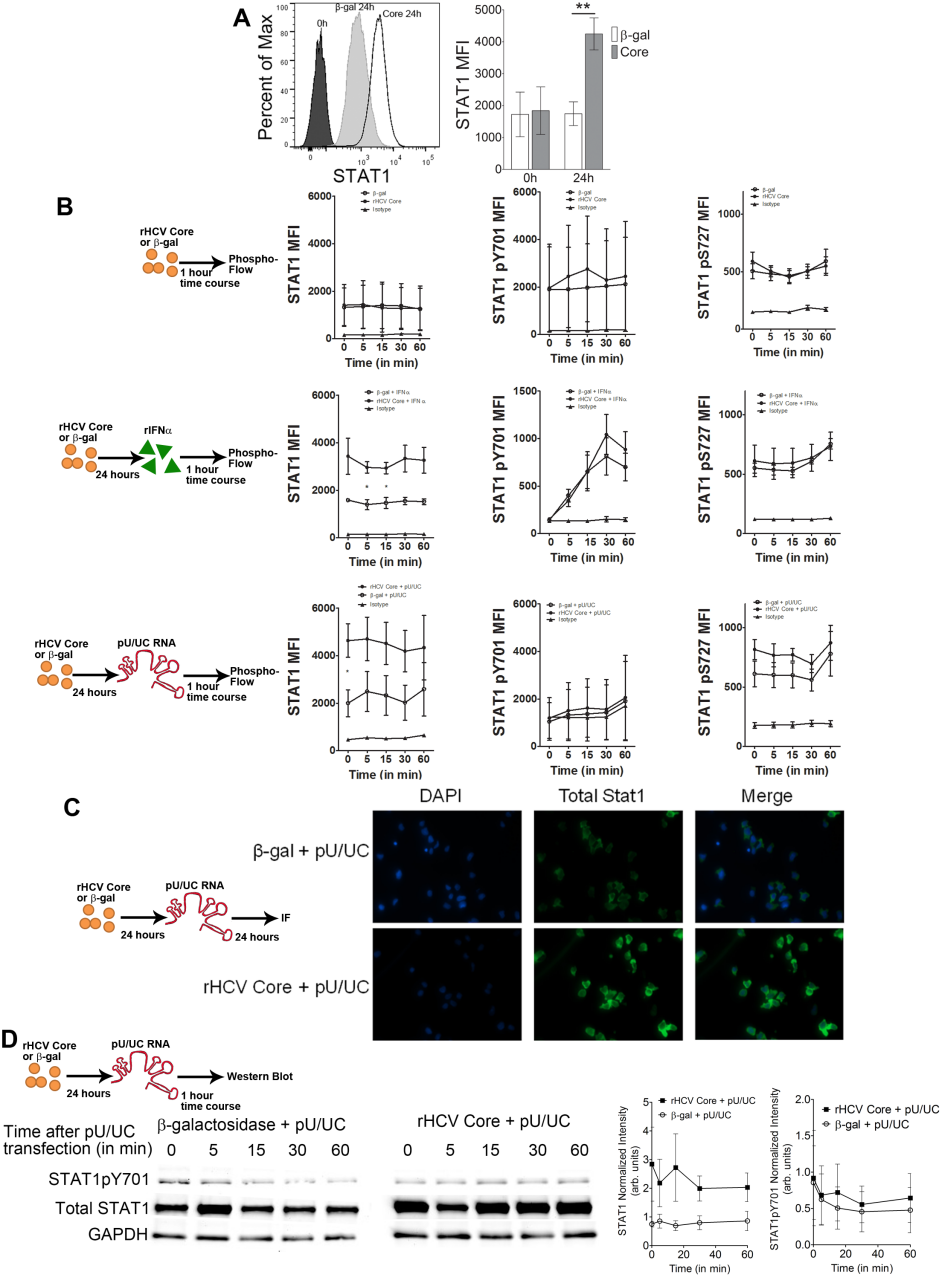
rCore alters JAK-STAT proteins. A) Representative histograms (left) and MFI (right) of STAT1 from 0 and 24 hours of rCore/β-gal protein exposure. B) MFI graphs of phosphoflow for STAT1 (left), STAT1pY701 (middle) and STAT1pS727 (right) after treatment with rCore/β-gal (top row), rCore/β-gal for 24 h followed by IFNα (100 ng/mL) stimulation (middle row) or rCore/β-gal for 24 h followed by pU/UC RNA stimulation (bottom row). C) Immunofluorescence (IF) showing STAT1 in rCore/β-gal pretreated cells followed by pU/UC RNA stimulation. Green – Total STAT1 Blue – nuclei. D) STAT1 and STAT1pY701 shown by Western Blot. Cells were treated for 24 hours with rCore/β-gal then stimulated with pU/UC RNA over time. Normalized densitometry shown on right. Representative blots, images and flow plots are shown. Graphs show combined data for 3 independent experiments. P values are results of Mann-Whitney comparison of the dots or bars indicated. *p<0.05 **p<0.01. Mean +/− SEM.

Since IFN production and IRF-7 levels were decreased in rCore exposed pDCs, we posited that this was due to decreased IFN signaling. As such, we examined the JAK-STAT signaling pathway, by measuring levels of total and phosphorylated STAT1 (Tyr701 and Ser727). STAT1 protein was induced in pDCs after 24 hours of rCore exposure (**Figure 3A**). However, 1 hour exposure of pDCs to rCore or β-gal had no effect on STAT1 total, Tyr701, or Ser727 proteins (**Figure 3B** **Top row**). rCore did not affect IFNα induced STAT1 Tyr701 and Ser727 phosphorylation despite the clear rCore-induced increase in total STAT1 (**Figure 3B** **Middle row**). Moreover, phosphorylation of STAT3 at Ser727 was intact in these conditions (**Figure S3B**). Similarly, rCore did not induce nor modulate STAT1 Tyr701 and Ser727 during HCV pU/UC RNA exposure, despite the clear rCore induced increase in total STAT1 (**Figure 3B** **bottom row**). There was a slight increase in the levels of STAT3pS727 after rCore pretreatment followed by pU/UC RNA stimulation (**Figure S3B**). Immunofluorescence further confirmed that STAT1 protein levels were increased upon exposure to rCore but not β-gal (**Figure 3C**). Finally, using Western blot, we confirmed that rCore induced total STAT1 and that STAT1 phosphorylation was not induced by pU/UC stimulation after pretreatment with rCore or β-gal (**Figure 3D**).

Together, these data show that extracellular rCore disrupts IFN signaling through a dysfunctional increase in non-phosphorylated STAT1. rCore treatment does not prevent IFNα induced STAT1 phosphorylation (**Figure 3B** **, middle row, middle column**), suggesting that upstream kinases such as Jak and Tyk are intact following exposure to rCore.

## Discussion

In an uninfected setting, pDCs are the main producer of type I IFNs, synthesizing up to 10^9^ IFN molecules per cell within 12 hours after activation [36]. However, despite robust pDC production of IFNα/β/λ following sensing of viral RNA *in vitro*, the majority of HCV-infected individuals remain persistently infected [37]. Here, we demonstrate that Core, known to circulate in high levels in chronically infected patients, is internalized by pDCs, and is associated with decreased IFN production following stimulation (**Figure 1**). Using published data [17], [24], we calculated the amount of HCV Core available to pDCs in HCV infected patients. Given the reduction in pDC number and the maximum reported amounts of Core from an infected patient, at most, there is 2.13 pg of Core per circulating pDC. Our system that used 10 pg/pDC is only slightly higher than the estimate based on published data. While these levels may not be physiologically accurate, this study provides proof of principle that HCV Core can inhibit IFN production from pDCs. In addition, while HCV Core is usually bound to antibody during later stages of chronic infection, early stages of acute infection have a window period that is characterized by the presence of HCV RNA, but a lack of anti-HCV antibodies[38]. It is during this window period that pDCs could be exposed to free circulating HCV Core that would influence the IFN response. Later on, after the development of the anti-HCV Core antibodies, the pDCs may already be reduced in numbers and function as previously described [8], [17], [19] as thus may not be affected as notably by HCV Core. We have recently shown that pDC express TLR-2 [21] and gC1qR (**Figure S4**)., known receptors for Core. Notably, gC1qR is a physiological inhibitor of RIG-I mediated antiviral responses [39]. In HEK293T cells, virus infection causes mature gC1qR to migrate to the mitochondria where it associates with MAVS and thus prevents RIG-I/MDA-5 from interacting with this adaptor molecule [39]. As pDCs are capable of acquiring HCVcc particles [40] and do not require virus infection for other innate immune activation [16], the presence of virions and viral proteins may be sufficient to activate gC1qR. Thus, activation of gC1qR may lead to an inhibition of RLR-induced IFN production following pU/UC RNA treatment. Our novel results provide insights into counter-regulatory mechanisms utilized by HCV to attenuate antiviral responses.

Prior work had suggested that IFN-α production by pDC was indirectly impaired [8]. Furthermore, HCV has been shown to inhibit pDCs through E2/BDCA-2 interactions [20]. In contrast, we found that Core directly reduced IFN production in GEN2.2-pDCs (**Figure 1**). Both mechanisms may be working in concert to attenuate innate immunity. We explored the mechanisms whereby core might be mediating this effect and found that it did not affect cell death or basal IFN levels (**Figure S1**). We examined expression of IRF-3 and IRF-7, finding that IRF-7 protein was decreased in rCore-treated pDCs that were transfected with the pU/UC RNA (**Figure 2C**). Additionally, pIRF-3 levels were unchanged suggesting that rCore does not inhibit the phosphorylation of IRF-3 (**Figure 2D**). Given that the IRF-7 levels after 24 hours of rCore or β-gal treatment remained the same, these data also suggest that the decrease of IRF7 shown in **Figure 2C** might be the consequence of lower levels of IFN production rather than a direct effect of Core (**Figure 2A**). Indeed, rCore treatment did not affect IRF7 protein levels when stimulated with IFN-α (**Figure 2A**), which may account for the observation that IFN-α treatment in acute HCV infection is associated with higher rates of immune restoration and viral control [41]. Additionally, using phosphoflow we determined that AKT and ERK were not phosphorylated in cells treated with rCore (**Figure S3C and S3D**), indicating that other innate signaling pathways are not affected by rCore.

STAT1 is the best characterized transcription factor known to regulate many of the biological effects mediated by IFNs in response to viral infections *in vivo* [42]. Accordingly, STAT1-deficient (STAT1^−/−^) mice are highly susceptible to viral infections due, in part, to lack of direct antiviral defense mediated by type I IFNs [42]. Therefore, it might seem a bit counterintuitive that rCore increased expression of non-phosphorylated STAT1 yet decreased IFN production from human pDC (**Figure 3**). However, we are confident in this finding as it was confirmed by multiple approaches. It is possible that STAT1 induction competes with other JAK/STAT events downstream of IFN receptor engagement [43], [44], as indicated recently in natural killer (NK) cells [45]. NK cells in HCV infection demonstrate increased STAT1 levels and impaired Type II IFN production as compared to uninfected controls [45]. Moreover, HCV infection of immortalized human hepatocytes has been to increase the expression of total STAT1 [46] while studies in hepatoma cell lines showed that Core binds STAT1 directly to prevent phosphorylation [30]. Furthermore, other post-translational modifications such as sumoylation inhibit activated STATs [47].

**Figure 4:**
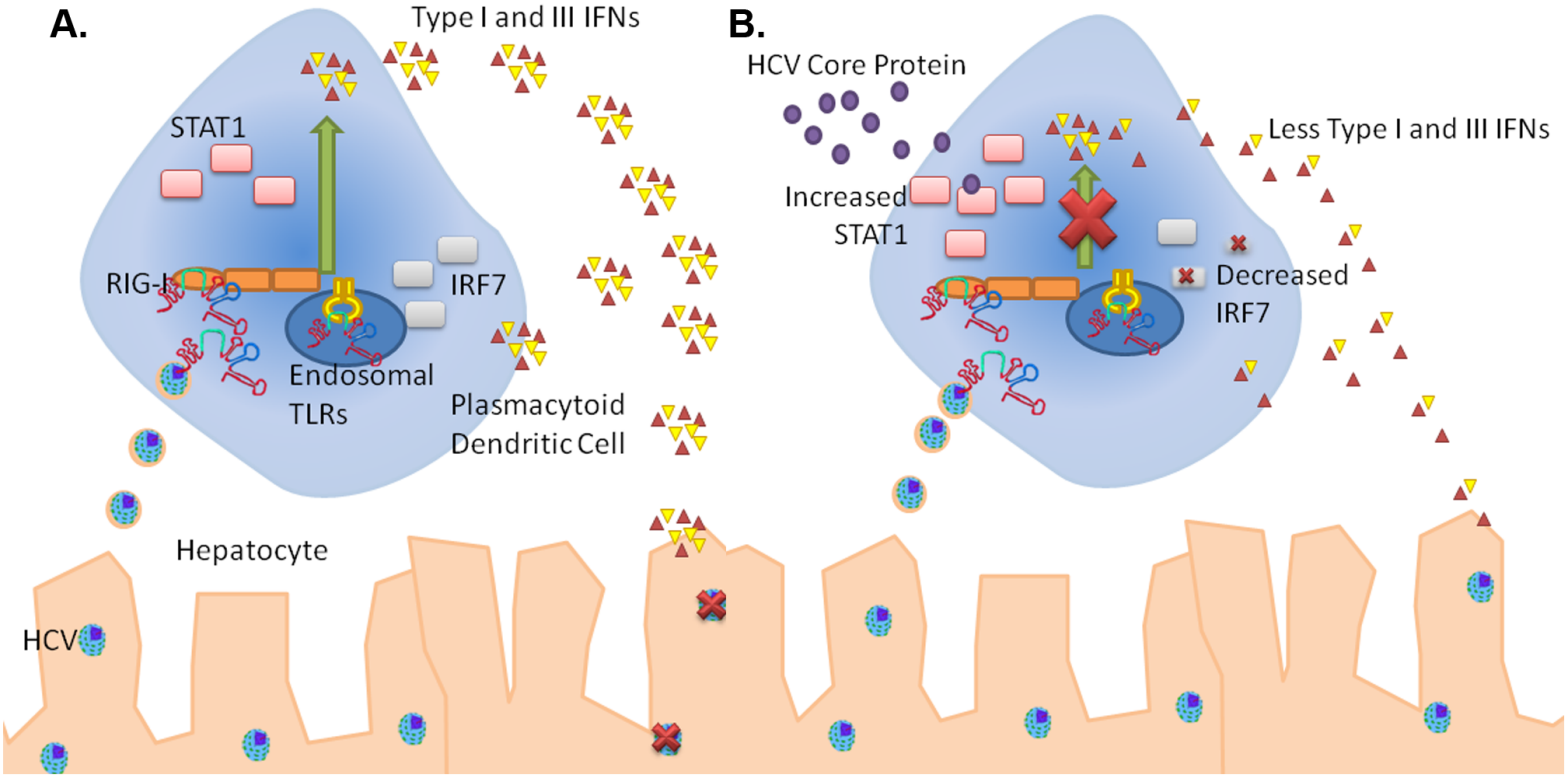
Paradigm Model of HCV Core acting on pDCs. A) pDCs respond to TLR stimulation and HCV PAMP to produce IFNs Type I and IFNLs. However, in the presence of HCV core (B), there is increased STAT1 but decreased IFNs production. The decreased IFN results in decreased IRF-7, which is an ISG.

In summary, this study shows that Core directly inhibits production of IFNα/β/λ within human pDC and dysregulates IRF-7 and STAT1 expression, possibly contributing to the persistent HCV infection (**Figure 4**). Thus, blockade of extracellular HCV Core protein may represent a novel therapeutic strategy for the enhancement of current treatments of HCV, particularly in those patients with acute HCV infection.

## Supporting Information

Figure S1 **rCore Alone Does Not Induce IFN Gene Upregulation or cell death.** A) Gene fold increases following treatment of GEN2.2-pDCs with rCore for 24 hours. B) Gene fold increases in IFN mRNA following pretreatment with GEN2.2-pDCs with 10 ng/mL LPS for 24 h then HCV PAMP RNA for 4 hours. C) Flow plots of GEN2.2-pDCs stimulated with rCore/β-gal. Top: Gates indicate Apoptotic cells (7-AAD^+^ and Annexin V^+^). Bottom: Gates indicate cells with activated caspase-3. D) CFSE plots of GEN2.2-pDCs treated with rCore/β-gal. No Protein – left panel, β-gal – middle panel, rCore – right panel. E) Immunofluorescence (40X) of GEN2.2-pDCs stained for nuclei (DAPI; blue) and Core (green). Combined data for 3 independent experiments (A & B); representative flow plots from 3 independent experiments (C & D). p values are results of Mann-Whitney comparison of the bars indicated. **p<0.01. Mean +/− SEM.
(TIF)

Figure S2 **IRF7 phosphorylation is not influenced by rCore.** Top – Schematic of experimental design. Phosphoflow of IRF-7 and IRF-7pS477/pS479 after 24 h of rCore/β-gal treatment followed by HCV pU/UC RNA treatment. Graphs of MFI of IRF7 (left) and pIRF7 (right) with combined data for 3 independent experiments. Mean +/− SEM.
(TIF)

Figure S3 **Phosphorylation of select signaling molecule is not influenced by rCore.** A) Experimental design. B–D) STAT3pS727(B), pAKT (C) and pERK1/2(D) MFI after treatment with rCore/β-gal (top row), rCore/β-gal for 24 h followed by IFNα (100 ng/mL) stimulation (middle row) or rCore/β-gal for 24 h followed by pU/UC RNA stimulation (bottom row). Graphs show combined data for 3 independent experiments. Mean +/− SEM.
(TIF)

Figure S4 **GEN2.2-pDCs express gC1qR.** Flow histogram demonstrating that GEN2.2-pDCs express gC1qR (CD93w), a reported receptor for HCV Core, on the cell surface. Representative flow histogram from 3 independent experiments.
(TIF)
