# Supplementary figures and images for: Hepatitis C Virus Core Protein Inhibits Interferon Production by a Human Plasmacytoid Dendritic Cell Line and Dysregulates Interferon Regulatory Factor-7 and Signal Transducer and Activator of Transcription (STAT) 1 Protein Expression
Source: PLoS One. 2014 May 1;9(5):e95627. doi: 10.1371/journal.pone.0095627 (PMC4006833; doi:10.1371/journal.pone.0095627)

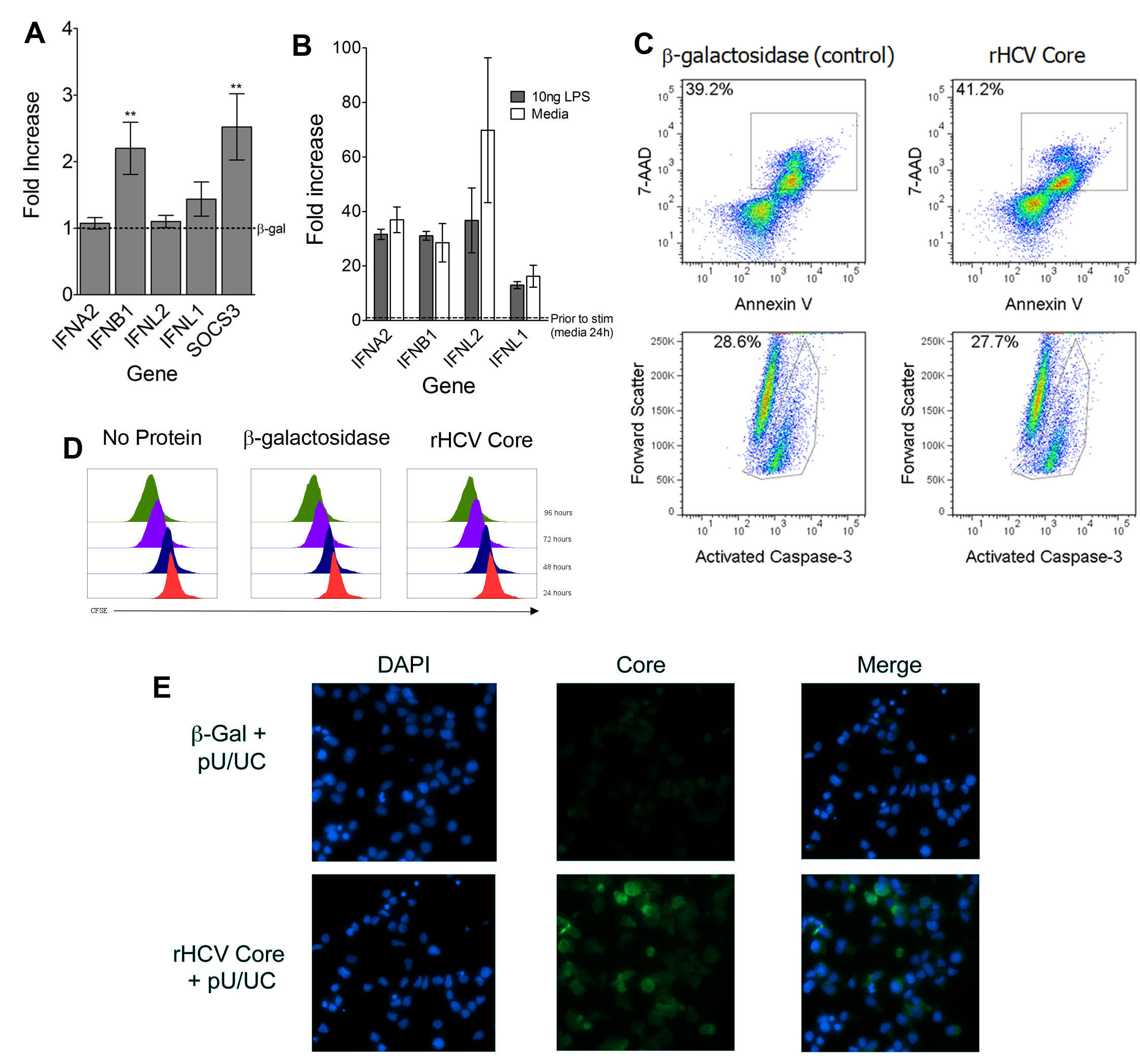

Supplement: Figure S1 — rCore Alone Does Not Induce IFN Gene Upregulation or cell death. A) Gene fold increases following treatment of GEN2.2-pDCs with rCore for 24 hours. B) Gene fold increases in IFN mRNA following pretreatment with GEN2.2-pDCs with 10 ng/mL LPS for 24 h then HCV PAMP RNA for 4 hours. C) Flow plots of GEN2.2-pDCs stimulated with rCore/β-gal. Top: Gates indicate Apoptotic cells (7-AAD+ and Annexin V+). Bottom: Gates indicate cells with activated caspase-3. D) CFSE plots of GEN2.2-pDCs treated with rCore/β-gal. No Protein – left panel, β-gal – middle panel, rCore – right panel. E) Immunofluorescence (40X) of GEN2.2-pDCs stained for nuclei (DAPI; blue) and Core (green). Combined data for 3 independent experiments (A & B); representative flow plots from 3 independent experiments (C & D). p values are results of Mann-Whitney comparison of the bars indicated. **p<0.01. Mean +/− SEM. (TIF) [file pone.0095627.s001.tif]

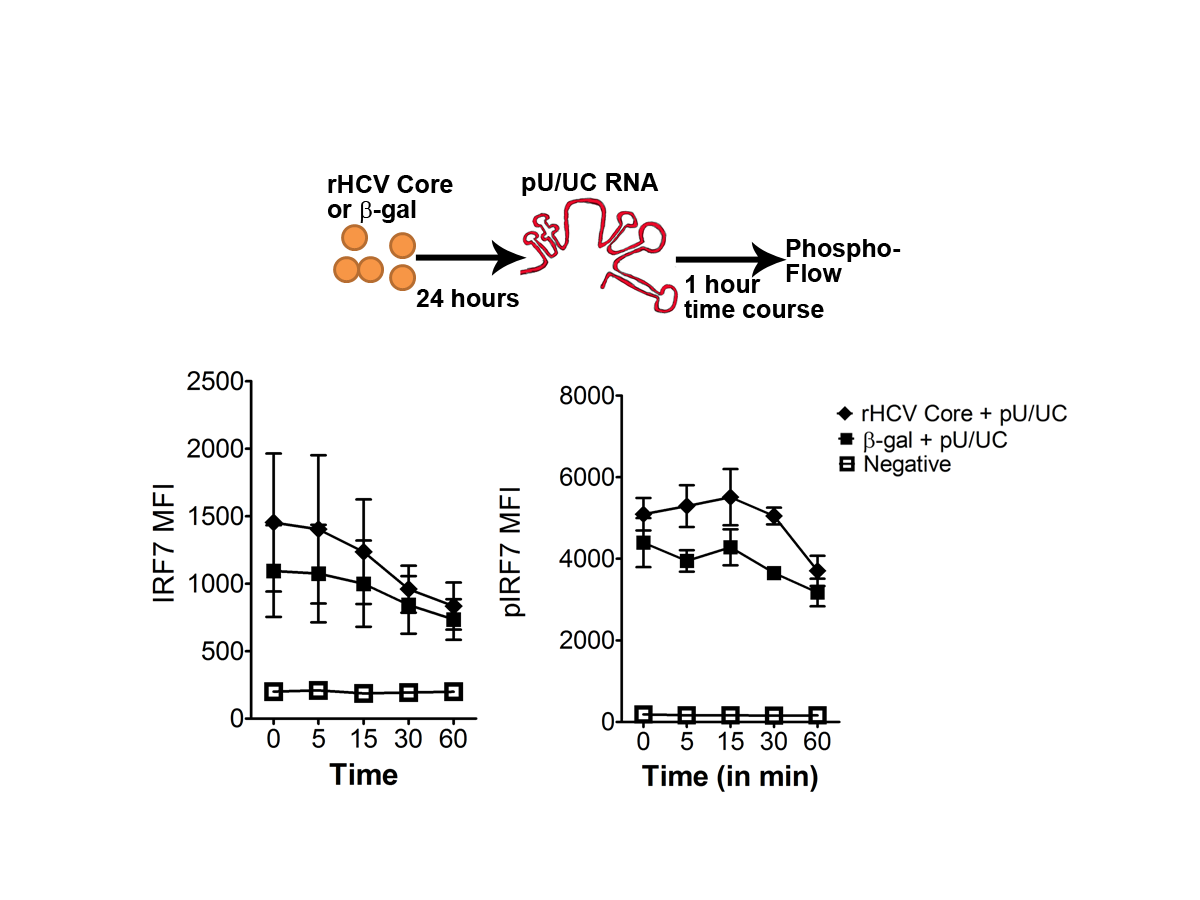

Supplement: Figure S2 — IRF7 phosphorylation is not influenced by rCore. Top – Schematic of experimental design. Phosphoflow of IRF-7 and IRF-7pS477/pS479 after 24 h of rCore/β-gal treatment followed by HCV pU/UC RNA treatment. Graphs of MFI of IRF7 (left) and pIRF7 (right) with combined data for 3 independent experiments. Mean +/− SEM. (TIF) [file pone.0095627.s002.tif]

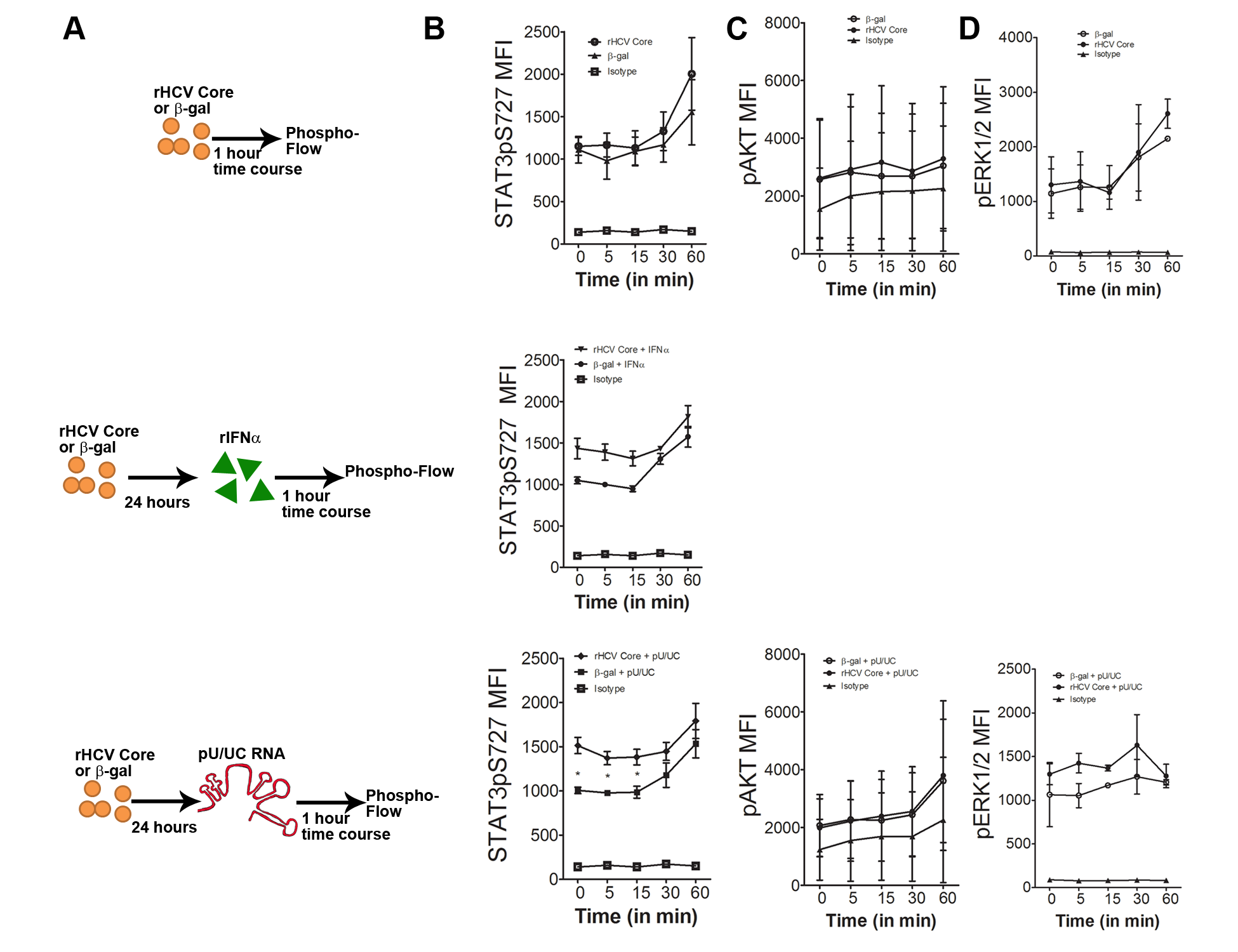

Supplement: Figure S3 — Phosphorylation of select signaling molecule is not influenced by rCore. A) Experimental design. B–D) STAT3pS727(B), pAKT (C) and pERK1/2(D) MFI after treatment with rCore/β-gal (top row), rCore/β-gal for 24 h followed by IFNα (100 ng/mL) stimulation (middle row) or rCore/β-gal for 24 h followed by pU/UC RNA stimulation (bottom row). Graphs show combined data for 3 independent experiments. Mean +/− SEM. (TIF) [file pone.0095627.s003.tif]

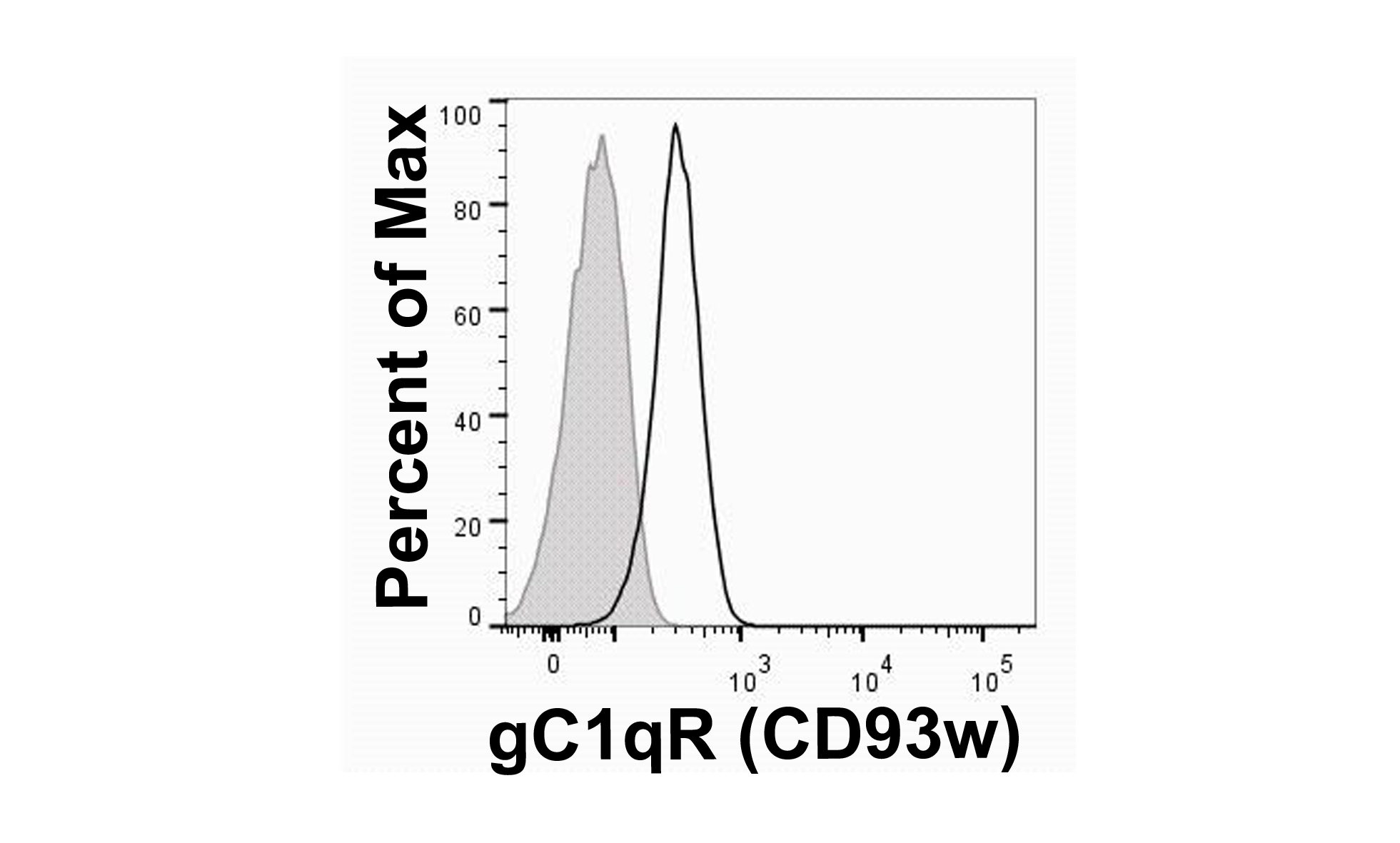

Supplement: Figure S4 — GEN2.2-pDCs express gC1qR. Flow histogram demonstrating that GEN2.2-pDCs express gC1qR (CD93w), a reported receptor for HCV Core, on the cell surface. Representative flow histogram from 3 independent experiments. (TIF) [file pone.0095627.s004.tif]
